# The Effect of Kangaroo Mother Care (KMC) on Breast Feeding at the Time of NICU Discharge

**DOI:** 10.5812/ircmj.2160

**Published:** 2013-04-05

**Authors:** Mohammad Heidarzadeh, Mohammad Bagher Hosseini, Mashallah Ershadmanesh, Maryam Gholamitabar Tabari, Soheila Khazaee

**Affiliations:** 1 Neonatal Health Office, Ministry of Health, Tehran, IR Iran; 2 Department of Pediatrics, Tabriz Medical Science University, Tabriz, IR Iran; 3 Neonatal Research Center, Tabriz Medical Science University,Tabriz, IR Iran; 4 Department of Midwifery, Islamic Azad University of Sari, Sari, IR Iran

**Keywords:** Breast Feeding, Kangaroo-Mother Care Method

## Abstract

**Background:**

Exclusive breastfeeding is one of the most important essential components of Kangaroo Mother Care.

**Objective:**

This study was performed to evaluate the effects of KMC on exclusive breastfeeding just at the time of discharge.

**Patients and Methods:**

In this cross sectional study, 251 consecutive premature newborns admitted to neonatal intensive care unit (NICU) between May 2008 and May 2009 in Alzahra University Hospital in Tabriz were evaluated. All of candidate mothers were educated for KMC method by scheduled program. Standard questionnaire was prepared by focus group discussion, and mothers filled it prior to infant hospital discharge.

**Results:**

In this study 157(62.5%) mothers performed kangaroo mother care (KMC group) versus 94 (37.5%) in conventional method care (CMC group). In KMC group exclusive breast feeding was 98 (62.5%) vs. 34 (37.5%), and P =.00 in CMC group, at the time of hospital discharge. Receiving KMC, and gestational age were the only effective factors predicting exclusive breastfeeding. Our result indicated that there was a 4.1 time increase in exclusive breastfeeding by KMC, and also weekly increase in gestational age increased it 1.2 times, but maternal age, birth weight, mode of delivery, and 5 minute Apgar score had no influence on it.

**Conclusions:**

KMC is more effective, and increases exclusive breast feeding successfully. It can be a good substitution for CMC (conventional methods of care). It is a safe, effective, and feasible method of care for LBWI even in the NICU settings.

## 1. Background

Kangaroo Mother Care for low-birth-weight infants was first started in Colombia in 1978. Edgar Rey in Bogotá, Colombia initiated what became known as Kangaroo Mother Care (KMC), as a response to both the lack of incubators, and the separation of mother and infant (1). A universally available, and biologically sound method of care for all newborns, but in particular for premature babies, with three components: 1) Skin-to-skin Contact 2) Exclusive breastfeeding 3) Support to the mother infant dyad (2). Kangaroo mother care is a skin–to–skin contact which is a part of revolution in premature infants care method (3) defined as continuous (as close to 24 h a day as possible) skin-to-skin contact between mother and her infant, ensured by placing infant in a strictly upright position on mother's chest (kangaroo position). Nutrition is based on (but not limited to) breast milk (4, 5). KMC can be started as soon as baby is stable, and receiving oral feeds. Babies with severe illness, and those requiring special treatment must wait until recovery before KMC can be started. Short KMC sessions can be initiated during recovery with ongoing medical treatment (Iv fluids, low concentration of additional oxygen) (6). Using kangaroo mother care is cost– effective, and has abundant advantages for mother, and infant (7). It causes better regulation of infant heartbeat. KMC babies have stable oxygen rates, and breathing. Breast milk production is stimulated by skin to skin care so baby gets all the benefits of breast milk including the correct milk for humans (2). It decreases infant crying (8). In a calm baby, food can be properly absorbed in the stomach, so the baby grows faster. The baby’s temperature stabilizes much faster on the mother’s chest than in an incubator (2, 9). KMC improves infant feeling by hearing mother heartbeat, touch, and visual contact with mother breast sucking, and mother odor (10, 11). Various studies have reported the advantages of using kangaroo mother care (KMC). Ramanathan reported that the number of mothers exclusively breastfeeding their babies at 6 week follow-up was double in the KMC group than the control group (12). Also, Suman found that exclusive breastfeeding improved in KMC group (98% versus 79%) in control group (13). This study was performed to evaluate the effects of KMC on exclusive breastfeeding just at the time of discharge. We believe that KMC is one of the vital indicators for monitoring and evaluation of neonatal intensive care units.

## 2. Objective

The aim of this study was to determine the association between KMC, and exclusive breast feeding

## 3. Patients and Methods

**Figure 1.**
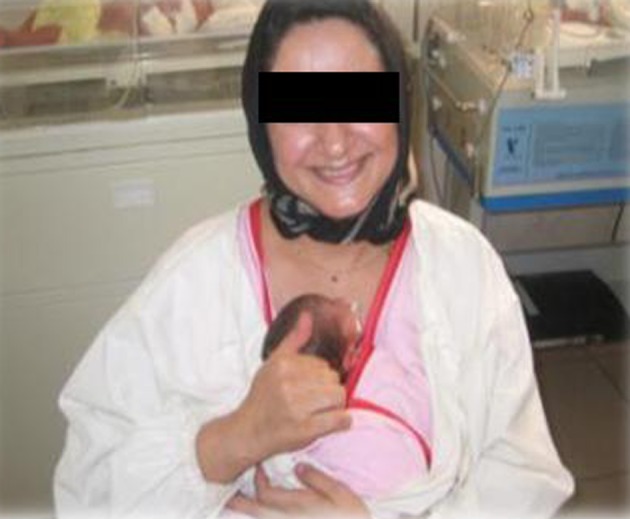
KangarooBag or Kanga Carrier Shirt

**Figure 2.**
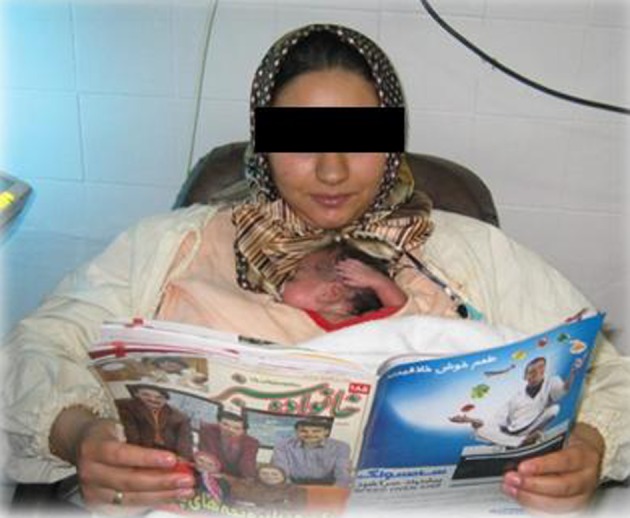
Mother Performs KMCWith Comfortable Situation

In this cross sectional study, 251 consecutive premature newborns admitted to neonatal intensive care unit (NICU) between May 2008 and May 2009 were evaluated. The present study was conducted in a Medical University Hospital "Alzahra Hospital” in Tabriz, State of eastern Azarbayjan, Iran, an excellent center for high-risk pregnancies, which was one of the first hospitals performed KMC as a routine care. Every mother that was healthy, and she and her baby had good condition, and had inclusion criteria were under evaluation. There was not any obligation to accept KMC performance. Mothers who performed KMC were included in KMC group, and those who did not were allocated for CMC group (conventional method care). A semistructured questionnaire, pregnancy history, and the socioeconomic status of the family were filled by mothers. Data about the infants’ feeding, and weight during hospital stay were collected from medical records. Inclusion criteria were as follows; 1) gestational age of newborn was at least 28 weeks, 2) newborn that breastfed or had parenteral nutrition, 3) newborn that used mechanical ventilation system with stable situation. Exclusion criteria were as follows; 1) newborn who had arterial cutter or chest tube, 2) Newborn who received vasodilator drug for regulate blood pressure, 3) newborn who had stage 3, and 4 intra ventricular hemorrhage. Training team included physician, and nurses of newborn intensive care unit (NICU) who had been trained enough in this field. Parents in KMC group should have been informed, trained, and supported every day. Educational topics were about kangaroo mother care method, and breast feeding. Mothers filled a chart to record number, and duration of care provided. If the mother was unable to fill that intensive care unit (NICU) staff were responsible to record information. In KMC group, mothers had to seat in a comfortable chair placed close to the baby's cradle. After the baby full feeds she provided kangaroo care between her breasts in a vertical or semivertical position, when the baby was not in KMC, the baby was placed in the cradle under heat lamp with adequately clothed, and covered. The babies in CMC group were managed in a cradle under heat lamps in NICU. During care, mothers should take off their blouse, and breast band. The mothers used a special suitable clothes called "kangaroo bag" or Kanga Carrier shirt which is designed to be safe for premature babies, and is designed for the first eight weeks of full term babies life before they have neck control. The shirt is convenient as it leaves both of the mother’s arms free to carry on with normal business of life, and walking around. The shirt is comfortable, and made from soft cotton. The Kanga Carrier is safe enough for mother, and baby to sleep together without fear of rolling on the baby, which is soothing for both mum and baby, so they get more rest (Figures 1 and 2).

During kangaroo mother care, newborns were not completely bare. Suitable clothing, and a soft hat were used to keep the newborns head warm (14). Duration of KMC was at least 1-3 hours which was repeated at least three times a day. If mothers were not tolerating this duration due to disease or special conditions, KMC was performed at least for 30 minutes every time. Mother daily bath and washing was recommended for preventing disease transmission. Mother should not have any contagious infectious diseases such as a common cold. Room temperature was between. For evaluating the effect of KMC on exclusive breastfeeding, newborns were classified into three groups based on their birth weight. 1) Weight under 1000g, 2) weight between 1000-1500g and 3) more than 1500g. And each group in KMC group was compared with the same group in CMC group. We performed a comparative analysis of the groups regarding the infants/ maternal characteristics and performing KMC, and type of feeding. Quantitative variables were analyzed with t test, chi-square test, and logistic regression. We considered KMC, and gestational age as independent variables, and exclusive breastfeeding, maternal age, birth weight, mode of delivery, and 5 minute Apgar score as dependent variables in logistic regression analysis. The statistical significance level was set at 0.05.

## 4. Results

**Figure 3.**
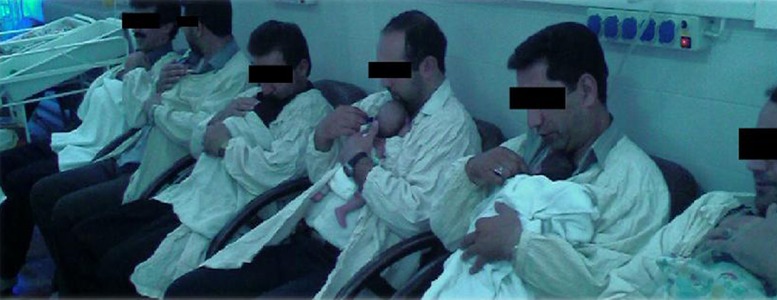
Fathers Are Doing Kangaroo Care at the NICU Ward

Table 1 shows the characteristics of 251 mothers, and their preterm infants in KMC group [157 (62.5%)] versus CMC group [94 (37.5%)] (Table 1). Average mother age, score at 5 minute Apgar, birth weight, and gestational age in preterm infant were similar in two groups (Table 1). In KMC group mothers had more vaginal delivery compared to those in CMC group. (43.8% vs. 25.3%) with a significant difference (P = 0.00). It shows that the tendency of mothers who had cesarean section delivery to do KMC were lower than those who had vaginal delivery. It may be due to the pain, and discomfort of section (Table 1). The average time for beginning KMC performance in the hospital was 7 days after birth. In KMC group 14 fathers helped mothers to do KC (Table 1) (Figure 3).

**Table 1.** Demographic, Clinical, and Characteristic in Mothers, and Preterm Infants

Variable	KMC^a^Group (n = 157)	CMC^a^Group (n = 94)	P value
**Maternal**			
Maternal age, Mean ± SD	27.75 ± 5.45	28.10 ± 6.03	0.48
Spontaneous vaginal delivery, No. (%)	64(43.8)	23(25.3)	0.00
5 minute Apgar scores, Mean ± SD	7.2 ± 1.7	7.1 ± 1.7	1.0
**Infant**			
**Birth weight, gr, Mean ± SD**	1754 ± 891	1811 ± 706	0.57
< 1000 gr; No. (%)	90(57.3)	38(40.4)	0.36
1000-1500 gr; No. (%)	53(33.8)	47(50)	0.06
> 1500 gr; No. (%)	14(8.9)	9(9.6)	0.60
**Gestational age in weeks, Mean ± SD**	32.08 ± 3.7	32.57 ± 3.7	0.31
**Start of KMC–day: Median (Mean)**	7(11.69)		
**Father KC**	14(9.7)		

^a^Abbreviation: CMC, Conventional method care; KMC, Kangaroo mother care

The group who practiced KMC, had more exclusive breast feeding at the time of hospital discharge than the mother who did not practice KMC (62.5% vs. 37.5%) with a significant difference (P = 0.00). Exclusive breast feeding was compared in newborns who were divided into 3 different weight groups. In the weight group less than 1000g exclusive breastfeeding in KMC group was (72.4% vs. 53%) in CMC group with a significant difference (P = 0.00). In the weight group between 1000-1500 g exclusive breastfeeding in KMC group was (42/8% vs. 24/4%) in CMC group with a significant difference (P = 0.00). In the weight group more than 1500g it was 3/7% vs. 3% with no significant difference (P = 0.63) respectively (Table 2).

**Table 2.** Comparison of Exclusive Breast Feeding Between KMC, and CMC Group at NICU Discharge

Birth Weight	KMC^a^Group, No. (%)	CMC^a^Group, No. (%)	P value
**> 1000gr**	71(72.4)	15(53.5)	0.00
**1000-1500gr**	12(42.8)	24(24.4)	0.00
**< 1500 gr**	3(3.06)	1(3.7)	0.63
**Total**	98(62.5)	34(37.5)	0.00

^a^Abbreviation: CMC, Conventional method care; KMC, Kangaroo mother care

The rate of artificial feeding in KMC group was less than control group (53.6% vs. 2.1%) with a significant difference (P = 0.00). (Table 3), also the rate of mixed feeding in KMC group was more than CMC group (35.4% vs. 8.9%) with a significant difference (P = 0.00) (Table 3).

**Table 3.** The kind of Infant Feedingat Hospital Discharge

Variable	KMC^a^Group, No. (%)	CMC^a^Group, No. (%)	P Value
**Exclusive breast feeding**	98(62.5)	34(37.5)	0.00
**Formula or Artificial feeding**	3(2.1)	42(53.6)	0.00
**Mixed feeding**	56(35.4)	8(8.9)	0.00

^a^Abbreviation: CMC, Conventional method care; KMC, Kangaroo mother care

Receiving KMC (Odds ratio; OR: 4.1; 95% CI: 2.2-7.5), and gestational age (OR: 1.21; 95%CI: 1.21-1.32) were the single effective factors to predict exclusive breast feeding probability. The result indicated that there was a 4.1 time increase in exclusive breast feeding by KMC (P = 0.00), and also every week increase in gestational age increased it 1.2 times (P = 0.00), but maternal age, birth weight, mode of delivery, and 5 minute Apgar score had not any influence on it (P > 0.05).

## 5. Discussion

The present study evaluated the effect of KMC performance on exclusive breast feeding in preterm infants at the time of hospital discharge. Our findings indicated that the mothers who performed KMC in NICU for their preterm infants had more exclusive breastfeeding at the time of hospital discharge (62.5% vs. 37.5%).%) than those who did not perform KMC. This result is similar with Suman (13), and Bicalho (15) study. Suman reported that mothers who had more KMC had more exclusive breastfeeding (98% versus 79%) (16), and Bicalho reported that the kangaroo units exhibited superior performance in relation to exclusive breastfeeding at discharge (69.2 vs. 23.8%). Brooks found that a group of newborns, who underwent KMC in (NICU), had 100% exclusive breastfeeding at the time of hospital discharge (17). The result of Boo study showed higher breastfeeding rate at discharge (29.7% vs. 14.5%) (16) Honorina reported that exclusive breastfeeding rates were higher in the kangaroo group at hospital discharge (82.6 vs. 0%) (18). There are only a few studies with methods and findings similar to our findings. The only study performed in Iran has different method compared to the present study. Kamalifard in her interventional study reported that mothers who started KMC immediately after birth for 60 minutes had more exclusive breast feeding duration at fourth month after birth (119.8 ± 13.27 vs. 110.75 ± 24.07). The method of this study was different with our study. Kamalifard evaluated full term infants that discharged after birth, and fallowed them during four months (19). In our study the mean age at commencement of KMC in NICU was 7 days. Babies with severe illness, and those requiring special treatment, could use KMC practice after recovery and stableness. Nagais, in his study showed that early east performance of KMC in the first 24 hours after birth, and late performance 24 hours after birth have no effect on mortality of the first 28 days after birth (20). Present study showed that 9.7% of fathers performed KC. Fathers or close relatives performing KC is helpfull for mother. Direct participation of fathers in child care ensures mothers , and leads to more motivation for them to continue KMC action (21),we could not find any similar study that report father kangaroo care but Kadam (22), and Cattaneo (23) reported that 64%, and 83% of husbands accepted KMC, and supported their wives respectively. The present study indicated that there was a 4.1 time increase in exclusive breast feeding by KMC at the time of hospital discharge. Honorina reported that infants who underwent KMC had a 2.34 time greater chance of being exclusively breastfed at discharge from hospital (18). Venancio showed that KMC is a protection factor for breastfeeding at discharge (24). It seems that kangaroo mother care (KMC) is an effective way to increase the exclusive breast feeding. KMC can be a good substitution for CMC (conventional methods of care). It is a safe, effective, and feasible method of care for LBWI even in the NICU settings.
